# MITOCHONDRIA, METABOLISM, AND GLUTATHIONE AS MEDIATORS OF COGNITIVE DECLINE IN AGING AND ALZHEIMER’S DISEASE

**DOI:** 10.1093/geroni/igae098.0806

**Published:** 2024-12-31

**Authors:** Rajagopal Sekhar, George Taffet

**Affiliations:** Chair; Discussant

## Abstract

Aging is the most important risk factor for cognitive decline and Alzheimer’s disease (AD), and age-related cognitive decline (ACD) is widely prevalent in older adults (OA). However, the mechanistic underpinnings of cognitive impairment in aging and AD remain poorly understood, and are a high-priority research focus toward finding solutions. Of all organs of the body, the brain requires a maximal amount of energy which is generated by mitochondria. Interruptions in energy availability could be critical to cognitive impairment in both ACD and AD. In parallel, defects in metabolism (glutathione/redox metabolism, glucose metabolism, inflammation, endothelial dysfunction) are also implicated as contributors to cognitive impairment. This symposium will discuss novel mechanistic underpinnings of cognitive decline in terms of glutathione deficiency, mitochondrial dysfunction and abnormalities in metabolism, discuss a novel intervention to reverse these defects in aging, and its applicability in AD. The symposium re will begin with a talk by Dr. Mahapatra on impaired mitochondrial energetics in patients with AD. Then Dr. Kumar will discuss cognitive decline in old mice and link it to underlying brain brain defects in glucose metabolism, mitochondrial impairment and glutathione deficiency, and present data on successfully reversing these defects with GlyNAC supplementation. Dr. Sekhar will discuss translation of GlyNAC supplementation to ACD and present results from a human trial showing improvements in cognition, glutathione deficiency, and novel defects in metabolism and mitochondria. As Discussant, Dr. Taffet will present data from 91 humans showing that glutathione deficiency as a key, novel defect in AD, and discuss symposium presentations.

